# Rapid and sensitive detection of bovine *Theileria annulata* parasite based on ERA-CRISPR/Cas12a technology

**DOI:** 10.3389/fmicb.2025.1647929

**Published:** 2025-08-04

**Authors:** Xiujuan Feng, Yongchang Li, Shiyun Li, Iqra zafar, Mohamed Abdo Rizk, Xianyue Fu, Zeyun Cui, Wei Zhang, Yang Zhang, Ercha Hu, Qingyong Guo, Bayin Chahan

**Affiliations:** ^1^Parasitology Laboratory, Veterinary College, Xinjiang Agricultural University, Ürümqi, China; ^2^Laboratory of Sustainable Animal Environment, Graduate School of Agriculture Science, Tohoku University, Sendai, Japan; ^3^Department of Internal Medicine and Infectious Diseases, Faculty of Veterinary Medicine, Mansoura University, Mansoura, Egypt

**Keywords:** enzymatic recombinase amplification, *Theileria annulata*, CRISPR Cas12a, enolase, molecular diagnostics

## Abstract

*Theileria annulata*, a globally significant blood parasite in livestock, causes substantial economic losses in resource-limited regions by compromising animal health and hindering the development of the livestock industry. To address this, a rapid, reliable, and sensitive diagnostic assay integrating enzymatic recombinase amplification (ERA) with CRISPR/Cas12a technology was developed. This assay enables visual interpretation through multiple detection modalities, including UV and blue light illumination. Among three primer pairs and two CRISPR RNA (crRNA) candidates screened, the F3/R3 primer set combined with crRNA1 demonstrated the best performance. The optimized ERA protocol achieved complete amplification within 20 min at 37°C. This assay exhibited high specificity for *T. annulata* detection, with a sensitivity limit of 10 copies/μL, a 100-fold greater sensitivity than conventional PCR, while completing detection within 40 min. Validation of 51 bovine blood samples from a farm in Turpan, Xinjiang, revealed that PCR detected 12 positive cases (23.5% prevalence), whereas the ERA-CRISPR/Cas12a system identified 15 positive cases (29.4% prevalence). The enhanced detection capability of this integrated method provides crucial technical support for field applications in resource-limited settings, effectively addressing the urgent need for rapid and accurate diagnosis of bovine theileriosis.

## 1 Introduction

*Theileria annulata* (*T. annulata*) is a tick-borne parasite; clinical symptoms primarily include fever, superficial lymphadenopathy, anemia, and respiratory distress (Ma et al., 2020). The disease occurs globally, and its high mortality rates and incidence notably impede livestock productivity in regions such as Southern Europe, North Africa, Central Asia, and East Asia, resulting in significant economic losses (Elati et al., 2024). The Xinjiang Uygur Autonomous Region, the largest province in China, is situated in the northwestern part of the country. Bordering eight countries, including Mongolia, Russia, Kazakhstan, and Pakistan. This region is characterized by vast deserts, mountain ranges, a continental climate distant from the ocean, and arid conditions. These geographic and climatic factors collectively foster an ideal environment for ticks and tick-borne diseases, contributing to the exceptionally high prevalence of *T. annulata* infections in Xinjiang (Guo et al., 2018; Li et al., 2023; Zhang et al., 2024). The symptoms of *T. annulata* infection are diverse, especially when infected animals exhibit fever, jaundice, and anemia. Such nonspecific manifestations can lead to misdiagnosis or missed diagnoses by healthcare professionals. Therefore, improving the diagnostic capabilities for theileriosis is essential.

During the past two decades, several diagnostic methods for *T. annulata* have been developed, including blood smear microscopy, PCR, real-time quantitative PCR (qPCR), loop-mediated isothermal amplification (LAMP) and ELISA (Al-Hosary et al., 2015; Cao et al., 2022; Kernif et al., 2024; Yasin et al., 2025). While ELISA provides rapid results and is considered the gold standard for diagnosing theileriosis-related diseases (Portanti et al., 2011), most of these methods either fail to determine the exact stage of the intraerythrocytic parasite or require expensive equipment, specialized technical expertise, or time-consuming procedures (Sinha et al., 2023; Ntesang et al., 2024). Therefore, there is an urgent need for rapid and accurate detection of *T. annulata* in clinical and field settings. Enzyme-driven recombinase isothermal amplification (ERA), a novel isothermal DNA amplification technique, offers a promising solution. ERA is highly efficient for field use due to its simple primer design, moderate temperature requirements (37–42°C), and ease of reagent storage (Li J. et al., 2024; Li X. et al., 2024). The method enables specific amplification of target DNA fragments within 10–20 min under isothermal conditions (Li J. et al., 2024; Li X. et al., 2024). Furthermore, advancements in result visualization—such as fluorescence-based, turbidity, colorimetric, or lateral flow assays—have simplified interpretation and enhanced practicality for field operations (Dhama et al., 2014; An et al., 2021; Wang et al., 2024). Su et al. (2025) developed a one-pot SHERLOCK assay for *Theileria annulata* detection using RPA and CRISPR-Cas13a. While their integrated approach reduces contamination risk from frequent lid opening, its minimum detection limit of 10^3^ copies/μL limits sensitivity. This lower sensitivity may result in false negatives during early-stage infections.

The CRISPR/Cas system is naturally present in approximately 40% of bacteria and 90% of archaea. Cas12a (formerly known as Cpf1), a programmable RNA-guided DNA nuclease classified within the Type V Class II CRISPR-Cas system, contains approximately 1,200–1,300 amino acids and functions as a single RNA-guided nuclease (Koonin et al., 2023; Badon et al., 2024). The integration of Cas12a with isothermal amplification technology enabled the incorporation of fluorophores and quenchers into oligonucleotide sequences at both termini (Tang et al., 2021). The mechanism begins with crRNA binding to the Cas12a protein, forming a Cas12a-crRNA complex that recognizes the T-rich protospacer adjacent motif (PAM). Subsequently, base-pairing between the crRNA and complementary single-stranded DNA (ssDNA) activates the enzyme’s non-specific ssDNA trans-cleavage activity, which produces a detectable fluorescence signal (Weng et al., 2023; Kadam et al., 2023). Currently, available detection methods for bovine *T. annulata* remain limited by insufficient sensitivity and subjective visual interpretation, particularly for on-site pathogen diagnosis. This limitation hinders early screening and effective disease control. To address this, we have developed a rapid, visual detection method for *T. annulata* using the ERA-CRISPR/Cas12a platform, specifically optimized for field applications. Improving the diagnostic accuracy for *T. annulata* could significantly enhance disease prevention and control efforts.

## 2 Materials and methods

### 2.1 Samples and reagents

15 Positive and 36 negative bovine *T. annulata* samples used in this experiment were collected from Turpan City, Xinjiang Uygur Autonomous Region, China. The methods for sample screening are traditional PCR and sequencing, and the primer sequences can be found in the reference (Zhyldyz et al., 2023).

### 2.2 Chemical reagents

Cas12a and its corresponding NEBuffer r2.1 were purchased from New England Biolabs (MA, USA). The 2 × Taq Master Mix, agarose gel recovery kit, and DNA purification kit were purchased from Beijing Tiangen Biochemical Technology Co., Ltd. (Beijing, China). The Basic ERA Kit (no. KS101) was purchased from GenDx Biotech Co., Ltd. (Jiangsu, China). PMD 19-T vector and DNA extraction reagents were purchased from Dalian Baosheng Biological Engineering Company (Dalian, China).

### 2.3 DNA extraction and construction of standard cloned plasmids

DNA extraction was performed following the instructions of the DNA extraction kit. Based on the GenBank database and the published sequence of the *enolase* gene (ENO) (GenBank accession: HQ646253.1), the enolase gene was amplified using primers already available in our laboratory. The sequences of ENO-F and ENO-R are shown in Supplementary Table S1. The *T. annulata enolase* gene was amplified from *T. annulata* positive-control DNA. The PCR reaction system and conditions can be found in Supplementary Figure S1 and the legend. PCR amplification products were analyzed by 1% agarose gel electrophoresis. The *T. annulata enolase* gene was then cloned into the PMD 19-T vector for sequencing validation, The sequencing results can be found in Supplementary data 3.1.

Plasmid extraction was performed using the TianGen Fast Plasmid Mini Kit, following the steps outlined in the instruction manual for bacterial cultures confirmed to be positive by sequencing. The concentration was then determined using a microvolume spectrophotometer, and the copy number was calculated using the formula: [Amount (copies/μL) = [DNA concentration (g/μL)/(plasmid length in base pair × 660)] × 6.02 × 10^23^]. The DNA samples were diluted in 1 × TE buffer to final concentrations ranging from 10^6^ to 10^0^ copies/μL and stored at −20°C as standard solutions until use.

### 2.4 ERA primer design

ERA primers were designed using Primer Premier 5.0 software (PREMIER Biosoft International, Palo Alto, CA, USA). following the manufacturer’s protocol (Suzhou GenDx Biotechnology Co., Ltd., Jiangsu, China).

### 2.5 Design of crRNA and ssDNA

Two crRNAs were designed according to crRNA primer design principles using the Liang Cpf1 online CRISPR design tool.^1^ The fluorescent reporter probe (ssDNA Reporter) is a short single-stranded DNA sequence with the 5′ end labeled with 6-carboxyfluorescein (6-FAM) and the 3′ end modified with black hole quencher-1 (BHQ1). The probe sequence was 5′(6-FAM)-TTATT-3′(BHQ1). Both crRNA and ssDNA were commercially synthesized and purified by Shanghai Shenggong Bioengineering Technology and Service Co., Ltd. (Shanghai, China).

### 2.6 Establishment and optimization of the ERA-CRISPR/Cas12a fluorescence system

The ERA-CRISPR/Cas12a system was established using a two-step method. The basic ERA kit was used to perform isothermal amplification of the *enolase* gene fragment from *T. annulata*. For the ERA reaction, 20 μL of rehydration buffer, 2.5 μL each of forward and reverse primers (10 μM), 21 μL of ddH_2_O, and 2 μL of template, were combined to prepare a 48 μL mixture. This premix was transferred to reaction tubes containing lyophilized enzyme pellets, followed by the addition of 2 μL of activator. The tubes were centrifuged briefly and incubated at 37°C for 20 min. The CRISPR/Cas12a detection system consisted of 2 μL ERA amplification product, 2 μL NEBuffer r2.1, 1 μL ssDNA (10 μM), 1 μL crRNA, 1 μL Cas12a, and 15 μL nuclease-free water. After thorough mixing, the mixture was incubated at 37°C for 20–30 min. Fluorescence signals were visualized by exposing the reaction tubes to blue light.

To optimize experimental conditions, parameters including ERA primer screening, reaction time, temperature, crRNA selection, and concentrations of ssDNA, Cas12a, and crRNA were systematically evaluated. A template concentration of 10^6^ copies/μL was used during optimization to determine optimal conditions.

### 2.7 *Theileria annulata*-ERA-CRISPR/Cas12a reaction time optimization

Enzyme free water served as negative control, and plasmids containing 10^6^ copies/μL were used as positive samples. ERA amplification products were mixed with Cas12a and incubated at 37°C for varying durations. Fluorescence images were captured at each time point, and fluorescence intensity was quantified using ImageJ software via grayscale analysis.

### 2.8 Sensitivity and specificity testing of ERA-CRISPR/Cas12a

The plasmid was serially diluted tenfold to concentrations ranging from 10^0^ to 10^6^ copies/μL, which served as the template for the ERA-CRISPR/Cas12a reaction. A negative control (nuclease-free water) was also included to determine the sensitivity of the method.

Pathogens such as *Babesia bovis*, *Anaplasma marginale*, *Rickettsia* sp., and *Trypanosoma evansi* cause clinical symptoms very similar to those of *Theileria annulata* infection, including anemia, fever, and emaciation. Notably, *Babesia bovis* and *Theileria annulata* are particularly difficult to distinguish microscopically. Therefore, to validate the specificity of our assay, we used these five pathogens. The established ERA-CRISPR/Cas12a method was applied to detect DNA from *Theileria annulata* and each of the other pathogens individually.

### 2.9 Detection of clinical samples

DNA was extracted from the collected bovine whole blood samples and analyzed using the ERA-CRISPR/Cas12a method. Results were validated against PCR-based sequencing to confirm their consistency. The optimized reaction system comprised: 250 nM Cas12 protein, 2 μL NEBuffer r2.1, 250 nM crRNA, 2 μL ERA product, 1,000 nM ssDNA, and nuclease-free DEPC water, with a final reaction volume of 20 μL. Cohen’s kappa statistics were calculated to compare agreement between the PCR and ERA/CRISPR-Cas12a methods using IBM SPSS Statistics (Version 27.0.1).

### 2.10 Statistical analysis

All data were analyzed using GraphPad Prism 9 (GraphPad Software, USA). Differences between groups were assessed using one-way analysis of variance (ANOVA). All experiments were repeated at least three times, and data are expressed as mean ± standard error. A *p*-value less than 0.05 was considered statistically significant.

## 3 Results

### 3.1 ERA-CRISPR/Cas12a-based visual rapid detection strategy

**Figure 1:**
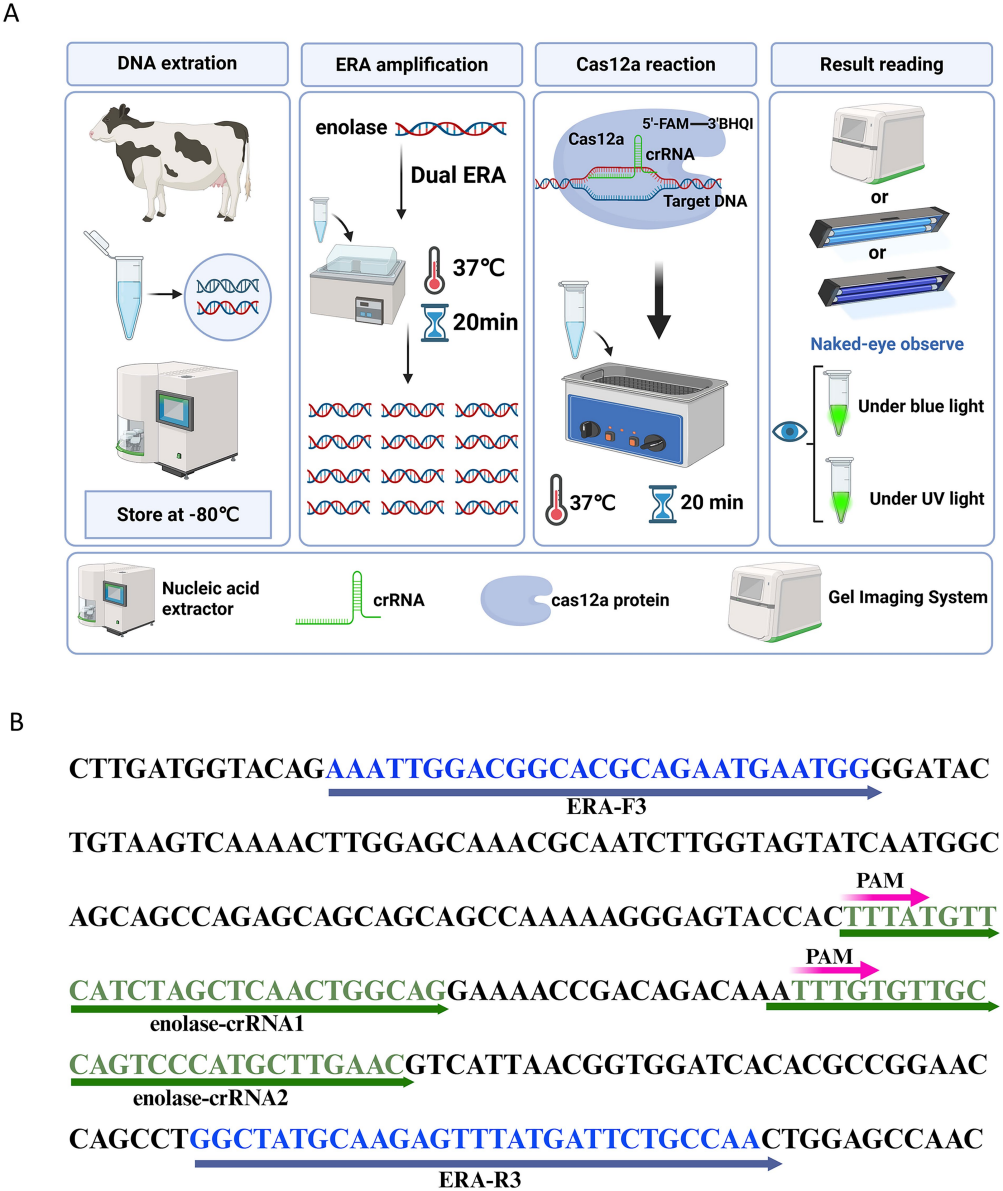
ERA-CRISPR/Cas12a workflow diagram. **(A)** Schematic workflow of the ERA-CRISPR/Cas12a assay platform. Genomic DNA is extracted from bovine blood, amplified by ERA, combined with a CRISPR/Cas12a mixture, and the results are visualized under blue/ UV light. **(B)** crRNA design for ERA-CRISPR/Cas12a detection.

**Figure 2:**
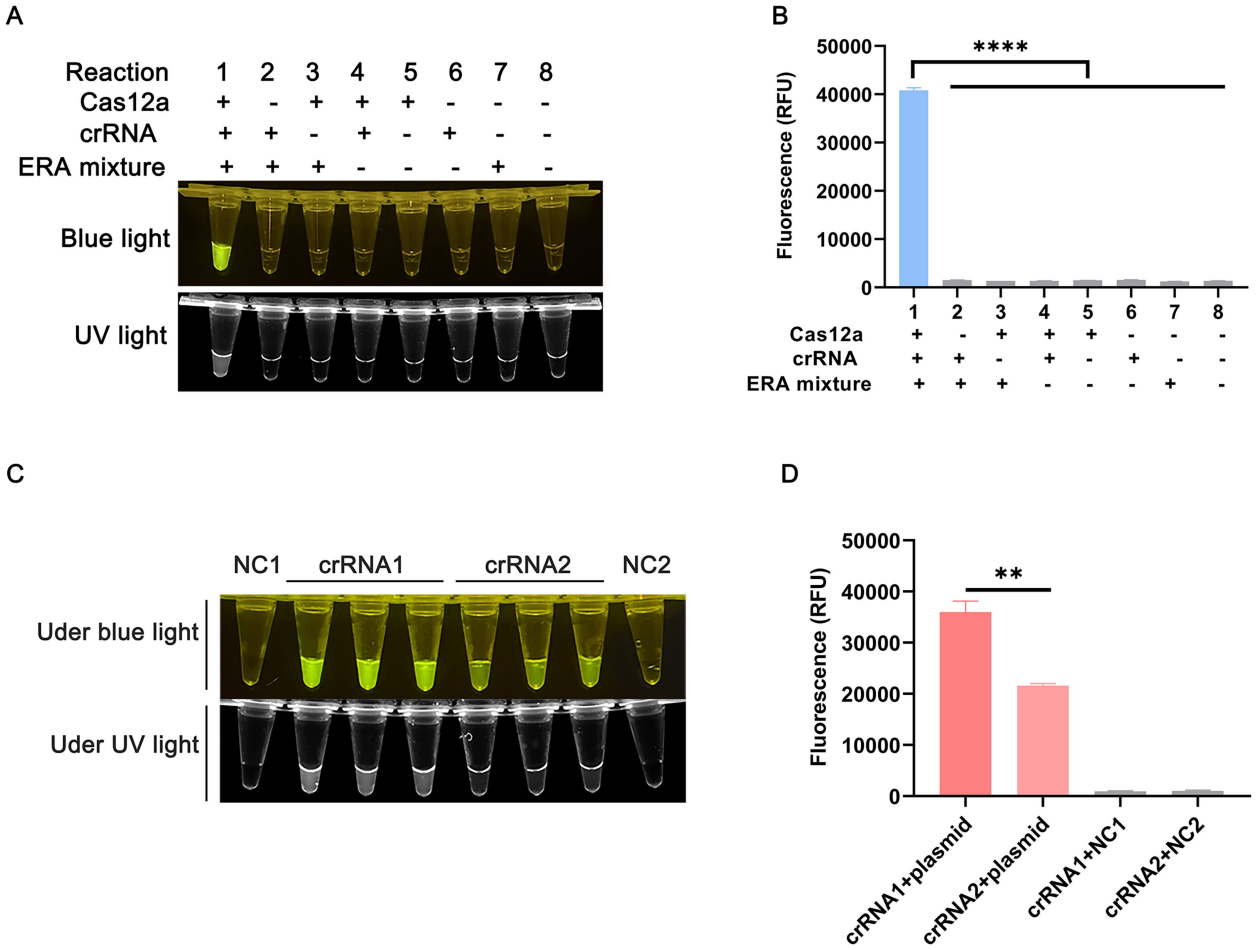
Establishment of the ERA-CRISPR/Cas12a assay platform. **(A)** Endpoint imaging of the Cas12a reaction system under blue/UV light. **(B)** Cas12a cleavage activity assay using plasmid with 10^6^ copies/μL as target. **(C)** Screening of crRNAs for detection of *T. annulata* under blue/UV light. NC1 is negative control for crRNA1; NC2 is negative control for crRNA2. **(D)** Graphical representation of the fluorescence intensity. All results were obtained from three independent experiments, and the values were displayed as mean ± SD. ****p < 0.0001 indicates statistical significance.

To reduce the detection time for *T. annulata* infection, we developed a rapid, efficient, and sensitive detection system by integrating enzymatic recombinase amplification (ERA) with the CRISPR/Cas12a system. The workflow of the ERA-CRISPR/Cas12a platform is illustrated in Figure 1A. Figures 2A,B present feasibility validation of the assay. All essential components – ERA amplification products, crRNA (Figure 1B), Cas12a, and ssDNA reporter – are required for fluorescence generation. Omission of any single component abolishes signal production, as demonstrated in Figures 2A,B. Results from the ERA-CRISPR/Cas12a assay were obtained within 20–30 min at 37°C, with the entire process requiring only 40 min. Detection outcomes were visually interpreted under blue light illumination.

### 3.2 ERA primers and crRNA screening

Three ERA primer pairs targeting the enolase gene were designed using Primer Premier 5.0 software (Supplementary Table S1). As shown in Supplementary Figure S2, the ERA-F3/R3 primer pair demonstrated optimal amplification efficiency without non-specific bands and was selected for subsequent experiments. Two crRNAs were designed within the ERA-F3/R3 amplicon (Supplementary Table S1; Figure 1B). Figure 2C shows that reactions using enolase-crRNA1 generated stronger fluorescence signals and clearer visual results (Figure 2D), leading to its selection as the optimal crRNA.

### 3.3 Optimization of ERA-CRISPR/Cas12a dual system

#### 3.3.1 Optimization of temperature and time for ERA reaction

**Figure 3:**
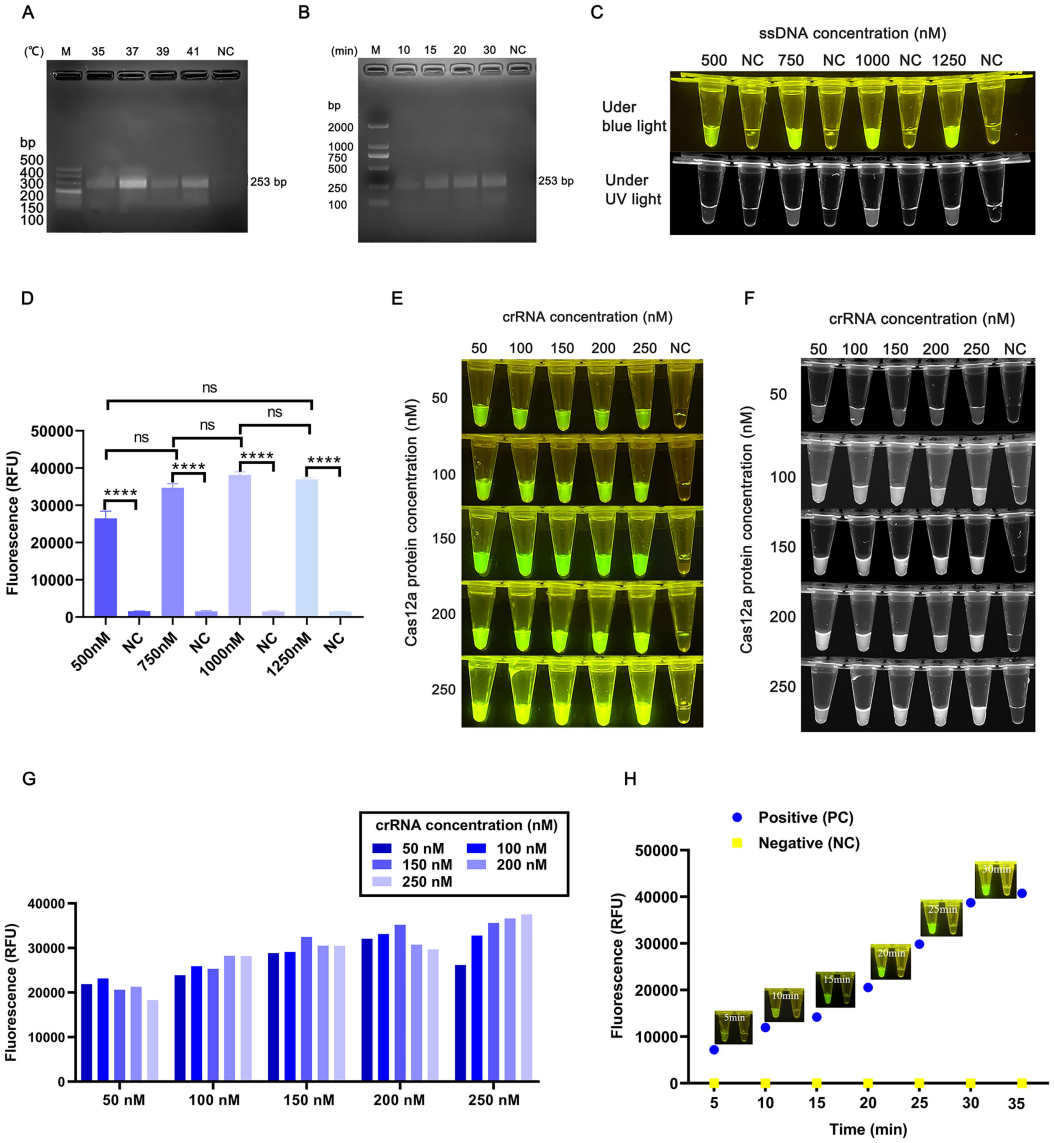
ERA-CRISPR/Cas12a assay optimization. **(A)** ERA reaction temperature screening using plasmids with a concentration of 10^6^ copies/μL. **(B)** ERA reaction time screening. **(C)** Fluorescence intensity analysis at different ssDNA concentrations; NC represents negative control. **(D)** Endpoint fluorescence data for ssDNA concentration screening. **(E)** Screening of crRNA and Cas12a protein concentrations under blue light. **(F)** Screening of crRNA and Cas12a protein concentrations under UV light. **(G)** Endpoint fluorescence analysis of crRNA and Cas12a protein concentration. **(H)** Optimization of Cas12a reaction time. Data are expressed as mean ± SD (*n* = 3). ****p < 0.0001 indicates statistical significance.

To maximize detection efficiency, the ERA and CRISPR/Cas12a systems were independently optimized. For the ERA system, amplification temperature and duration were systematically evaluated. Figure 3A indicates that the highest amplification efficiency occurred at 37°C. Figure 3B demonstrates that sufficient amplification products were obtained after 20 min of ERA reaction. To balance speed and efficiency, 20 min was selected as the optimal amplification time, resulting in optimized ERA protocol of 37°C for 20 min.

#### 3.3.2 Optimization of ssDNA concentration

To enhance signal clarity, ssDNA concentrations ranging from 500 nM to 1,250 nM were tested in Cas12a reactions using ERA amplification products. Figure 3C reveals that 1,000 nM ssDNA produced fluorescence intensity comparable to 1,250 nM (Figure 3D). Considering reagent cost, 1,000 nM ssDNA was selected for further experiments.

#### 3.3.3 Optimization of Cas12a and crRNA concentrations

Under these conditions, orthogonal experiments testing gradient concentrations of Cas12a protein and crRNA were conducted (Figures 3E,F). Fluorescence intensity was analyzed via blue light imaging and grayscale quantification using Image J software. As shown in Figure 3G, the optimal concentrations were determined to be 250 nM Cas12a protein and 250 nM crRNA.

### 3.4 Optimization of the reaction time for the ERA-CRISPR/Cas12a dual system

The ERA amplification products were mixed with Cas12a and incubated at 37°C. Fluorescence signals were detected after 5 min of incubation, with intensity gradually increasing over time (Figure 3H). No fluorescence was observed in the negative control. Grayscale analysis confirmed strong fluorescence signals at both 20 and 30 min. To minimize total detection time, 20 min was selected as the optimal reaction duration for the CRISPR/Cas12a system. Combined with the 20-min ERA amplification step, the total detection time for *T. annulata* using the integrated ERA-CRISPR/Cas12a platform was 40 min.

### 3.5 Specificity analysis of the ERA-CRISPR/Cas12a dual system

**Figure 4:**
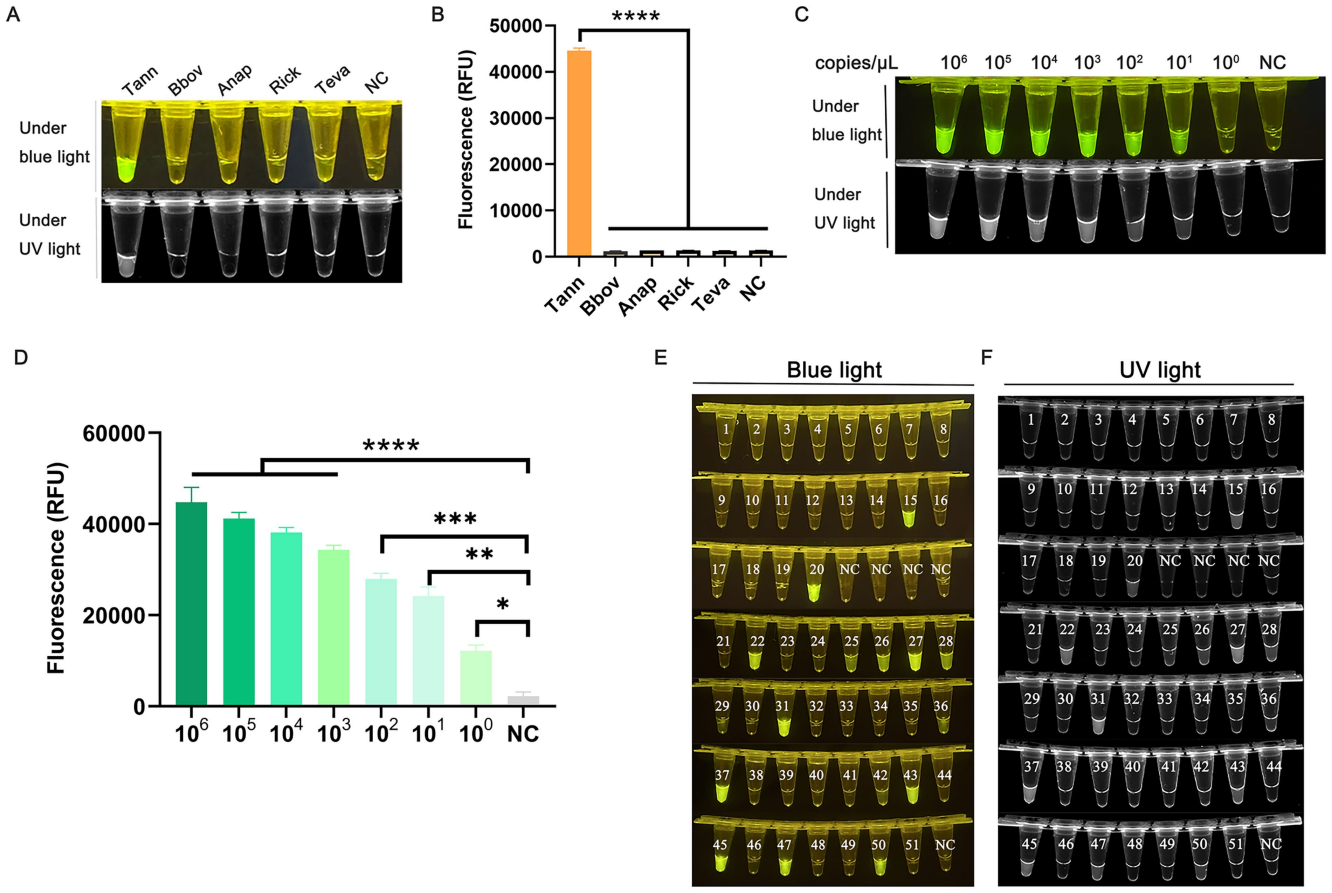
Specificity, sensitivity, and clinical validation of the ERA-CRISPR/Cas12a assay. **(A)** Specificity assessment using genomic DNA from non-target pathogens: *T. annulata* (Tann), *Babesia bovis* (Bbov), *Anaplasma marginale* (Anap), *Rickettsia* sp. (Rick), and *Trypanosoma evansi* (Teva). **(B)** Graphical representation of grayscale analysis of endpoint fluorescence valuesusing ImageJ and Graphpad Prism. **(C)** Sensitivity evaluation using serially diluted standard plasmid pMD-19-T-SBP2 (10^6^ to 10^0^ copies/μL). **(D)** Graphical representation of grayscale analysis of endpoint fluorescence values using ImageJ and Graphpad Prism. **(E,F)** Clinical validation using 51 bovine blood samples. Endpoint fluorescence images under UV/blue light are shown.

The specificity of the dual system was evaluated using four non-target DNA of bovine protozoan pathogens: *B. bovis*, *A. marginale*, *Rickettsia* sp., and *T. evansi* (Figure 4A). Only *T. annulata* exhibited a statistically significant increase in fluorescence intensity (Figure 4B). No amplification or fluorescence signals were detected in non-target DNA samples, confirming the high specificity of the ERA-CRISPR/Cas12a system.

### 3.6 Sensitivity analysis of the ERA-CRISPR/Cas12a dual system

For sensitivity testing, recombinant plasmids were serially diluted (10^6^ to 10^0^ copies/μL) and analyzed in parallel using both ERA-CRISPR/Cas12a and conventional PCR. The PCR limit of detection (LOD) was 10^3^ copies/μL (Supplementary Figure S3), whereas the ERA-CRISPR/Cas12a system achieved an LOD of 10 copies/μL (Figures 4C,D), indicating a 100-fold increase in sensitivity compared to PCR.

### 3.7 Clinical sample validation

Using the optimized ERA-CRISPR/Cas12a assay, we detected *Theileria annulata* in 15 of 51 bovine blood DNA samples (Supplementary Table S2; Figures 4E,F). Simultaneously, PCR analysis of the same samples identified 12 positives (Supplementary Table S3 and Supplementary Figure S4). According to the formula (Genders et al., 2012), we calculated the specificity and sensitivity of ERA/CRISPR Cas12a and PCR methods. The specificity and sensitivity of the ERA/CRISPR Cas12a method are both 100%; the specificity of PCR method is 100%, but the sensitivity is 80%. Cohen’s kappa coefficient (*κ* = 0.850, *p* < 0.001) indicated near-perfect agreement between the ERA/CRISPR-Cas12a and PCR methods, as values approaching 1 denote strong concordance (Supplementary Table S3).

## 4 Discussion

Theileriosis is one of the most economically significant diseases affecting livestock in tropical and subtropical regions, characterized by high morbidity and mortality rates in various cattle breeds (Gharbi et al., 2020). *T. annulata*, highly pathogenic tick-borne parasite in livestock, poses a major threat to livestock industry in northern and southern Xinjiang, China, as well as eight neighboring countries. Rapid detection of *T. annulata* is critical for controlling disease transmission and minimizing its agricultural and economic impact (Qi et al., 2018). Current diagnostic practices primarily rely on conventional microscopy, which often yields unreliable diagnoses due to low sensitivity and specificity. To address these limitations, this study targeted the enolase gene as a molecular marker to enhance the precision of theileriosis diagnostics. Results demonstrated marked upregulation of the enolase gene in *Theileria*-infected cattle, validating its utility as a molecular biomarker. Furthermore, ERA-CRISPR/Cas12a system improved detection sensitivity and specificity compared to traditional methods, providing a foundation for early diagnosis and targeted interventions.

Previous studies have shown that apicomplexan parasites lack aerobic metabolism, exclusively relying on anaerobic glycolysis for energy production (Piro et al., 2021). In this pathway, enolase catalyzes the conversion of 2-phosphoglycerate (2-PGA) into phosphoenolpyruvate (PEP), making the enzyme indispensable for the survival of *Theileria*. To explore this role, researchers conducted comprehensive experiments demonstrating that disrupting or inhibiting the enolase gene impaired glycolysis, reduced energy production, and suppressed *Theileria* proliferation (Yakarsonmez et al., 2020). This finding highlights enolase as a promising therapeutic target for anti-*Theileria* strategies. Building on this discovery, the enolase gene was leveraged to establish the ERA-CRISPR/Cas12a method, enhancing diagnostic precision for *T. annulata*. Additionally, using “DNAMAN V6,” the *T. annulata* enolase sequence (HQ646253) was compared against those of *Bos taurus* (281141), *Babesia bovis* (AK441830), *Anaplasma marginale* (7397988), and *Trypanosoma* sp. (NW_008825620) (Supplementary Figure S5). The exceptionally low sequence similarity with bovine enolase (4.24%) indicates minimal risk of host-derived false positives. Moderate similarities were observed with other pathogens: 62.29% with *B. bovis* consistent with shared Apicomplexan phylogeny and conserved glycolytic machinery; (Gardner et al., 2005), 52.69% with *A. marginale*, and 50.96% with *Trypanosoma* sp. These values reflect their distinct evolutionary origins (Proteobacteria and Kinetoplastida, respectively). The 38–49% sequence divergence from non-Theileria pathogens provides a molecular basis for our assay’s high specificity.”

Currently, the primary diagnostic methods for *T*. *annulata* include microscopy and PCR (Ullah et al., 2022). However, these techniques depend on specialized equipment and trained personnel, limiting their accessibility in resource-limited settings. ERA, an isothermal nucleic acid amplification technique, operates at 37°C-42°C without expensive instrumentation and has been applied to detect pathogens such as malaria parasites (Tavares et al., 2024), *Epstein–Barr virus* (Li et al., 2024), and *Mycoplasma pneumoniae* (Deng et al., 2022). Despite its advantages, ERA is susceptible to nonspecific amplification, requiring secondary signal amplification to ensure specificity. In the CRISPR/Cas12a system, crRNA enables sequence-specific target recognition, providing a secondary signal amplification step that improves detection specificity (Zeng et al., 2023).

Beyond its applications in gene editing, the CRISPR/Cas system demonstrates significant potential in pathogen nucleic acid detection and offers considerable advantages in molecular diagnostics (Xu and Li, 2020). However, the Cas12a system alone has inherent limitations in detection efficiency. By integrating ERA with CRISPR/Cas12a, we overcame these challenges and achieved substantially enhanced detection performance. The method demonstrated high specificity, with no cross-reactivity observed with hemoprotozoan infections such as babesiosis, trypanosomiasis, or anaplasmosis. Moreover, the ERA-CRISPR Cas12a platform achieved a detection limit of 10 copies/μL, showing much higher sensitivity compared to conventional PCR. Results could be visualized directly via fluorescence under blue light, making the method ideal for field use without specialized equipment.

Paliwal et al. (2020) developed a colorimetric loop-mediated isothermal amplification (LAMP) assay for detecting *T. annulata*. However, the LAMP assay requires four primers and operates at 60°C–65°C, increasing the likelihood of primer dimer formation and imposing stringent primer design and temperature requirements (Novi et al., 2025). In contrast, the ERA-CRISPR dual system operates at a constant reaction temperature of 37°C. In this study, comparative analysis of blood samples using ERA-CRISPR/Cas12a and PCR confirmed that ERA-CRISPR/Cas12a dual system is more sensitive, convenient, and efficient than PCR based methods.

Our findings underscore ERA’s advantages over traditional PCR in sensitivity, specificity, and speed, making it ideal for rapid diagnostics and field-based epidemiological studies. Compared to the RPA-CRISPR/Cas13a assay for *Theileria annulata* detection (Su et al., 2025) and the qPCR method (Ros-García et al., 2012), the ERA/CRISPR-Cas12a assay developed in this study achieved a significantly lower detection limit (10 copies/μL) and the fastest total detection time (40 min). However, repeated lid-opening and pipetting during the ERA-CRISPR Cas12a workflow risk aerosol contamination and false positives. In future research, in order to avoid cross contamination and aerosol contamination, we will consider adding reagents similar to dUTP (deoxyuridine triphosphate) during ERA amplification, Refer to Lai et al.’s (2022) method. Additionally, while the freeze-dried ERA enzyme retains stability under short-term ambient conditions (e.g., during transport or temporary storage), long-term storage at room temperature may lead to activity degradation. This represents a significant limitation for deployment in resource-limited settings. Future research will focus on protocol optimization, such as modifying Cas12a cleavage systems or integrating additional Cas proteins to enable one-tube detection method, thereby minimizing procedural complexity and contamination risks. We also aim to streamline sample processing by implementing crude DNA extraction techniques to enhance the practicality of the workflow. To further simplify field applications, we plan to replace ssDNA modifications with lateral flow test strips for visual detection, enabling rapid, equipment-free interpretation of results for cattle piroplasmosis.

## Data Availability

The original contributions presented in the study are included in the article/Supplementary material, further inquiries can be directed to the corresponding authors.

## Footnotes

^1^ http://bioinfolab.miamioh.edu/CRISPRDT/

## Supplementary material

SUPPLEMENTARY TABLE S1 Primer and probe sequences in this study.

SUPPLEMENTARY TABLE S2 Detection of *T.annulata* in clinical samples of bovine blood.

SUPPLEMENTARY TABLE S3 Cohen’s kappa coefficient.

SUPPLEMENTARY FIGURE S1 Standard plasmid electrophoresis results of *T. annulata*-enolase.

SUPPLEMENTARY FIGURE S2 Screening of 3 pairs of ERA primers.

SUPPLEMENTARY FIGURE S3 Sensitivity of PCR method.

SUPPLEMENTARY FIGURE S4 PCR detection results of bovine blood samples.

SUPPLEMENTARY FIGURE S5 Enolase gene sequence alignment results.

SUPPLEMENTARY FIGURE S6 ERA/CRISPR-Cas12a detection of 2-9 copies plasmid standards.

SUPPLEMENTARY Data 3.1 Sequencing results of plasmids

SUPPLEMENTARY Data 3.2 Observe the test results using a portable blue light lamp.

SUPPLEMENTARY Data 3.3 Observe the detection results in the blue light glue cutting instrument.

SUPPLEMENTARY Data 3.4 Observe the test results under the ultraviolet gel imager.
