# Supplementary material for: Rapid and sensitive detection of bovine Theileria annulata parasite based on ERA-CRISPR/Cas12a technology
Source: Front Microbiol. 2025 Aug 4;16:1647929. doi: 10.3389/fmicb.2025.1647929 (PMC12358460; doi:10.3389/fmicb.2025.1647929)

Supplementary Material

# 1 Supplementary Tables

**Table 1 Primer and probe sequences in this study**

| Name | Sequence (5'-3') |
| --- | --- |
| ENO-F | CAATTTTTAGATTCCAGAGG |
| ENO-R | CAGCTCTTCTTCGATGC |
| ERA-F1 | GTGTTAATAACAATTTTTAGATTCCAGAGG |
| ERA-R1 | GTCTCCATCTCGCAATTCTAGGGCTTCATA |
| ERA-F2 | AGATACCTTGATGGTACAGAAATTGGACGG |
| ERA-R2 | GCTAGATGAACATAAAGTGGTACTCCCT |
| ERA-F3 | AAATTGGACGGCACGCAGAATGAATGG |
| ERA-R3 | TTGGCAGAATCATAAACTCTTGCATAGCC |
| enolase-crRNA1 | UAAUUUCUACUAAGUGUAGAUCUGCCAGUUGAGCUAGAUGAACA |
| enolase-crRNA2 | UAAUUUCUACUAAGUGUAGAUUGUUGCCAGUCCCAUGCUUGAAC |
| ssDNA | FAM-TTATT-BHQ1 |
| PCR primers for clinical sample testing | F: TTCGTTATCTCATCCGTT R: ATACTTGGCATTGTTTGG |

**Table 2 Detection of *T.annulata* in clinical samples of bovine blood**

| No. | Species | Sample type | Detection results | |
| --- | --- | --- | --- | --- |
|  |  |  | ERA-CRISPR/Cas12a | PCR |
| 1 | bovine | blood | - | - |
| 2 | bovine | blood | - | - |
| 3 | bovine | blood | - | - |
| 4 | bovine | blood | - | - |
| 5 | bovine | blood | - | - |
| 6 | bovine | blood | - | - |
| 7 | bovine | blood | - | - |
| 8 | bovine | blood | - | - |
| 9 | bovine | blood | - | - |
| 10 | bovine | blood | - | - |
| 11 | bovine | blood | - | - |
| 12 | bovine | blood | - | - |
| 13 | bovine | blood | - | - |
| 14 | bovine | blood | - | - |
| 15 | bovine | blood | + | + |
| 16 | bovine | blood | - | - |
| 17 | bovine | blood | - | - |
| 18 | bovine | blood | - | - |
| 19 | bovine | blood | - | - |
| 20 | bovine | blood | + | + |
| 21 | bovine | blood | - | - |
| 22 | bovine | blood | + | + |
| 23 | bovine | blood | - | - |
| 24 | bovine | blood | - | - |
| 25 | bovine | blood | + | - |
| 26 | bovine | blood | + | + |
| 27 | bovine | blood | + | + |
| 28 | bovine | blood | + | + |
| 29 | bovine | blood | - | - |
| 30 | bovine | blood | - | - |
| 31 | bovine | blood | + | + |
| 32 | bovine | blood | - | - |
| 33 | bovine | blood | - | - |
| 34 | bovine | blood | - | - |
| 35 | bovine | blood | - | - |
| 36 | bovine | blood | - | - |
| 37 | bovine | blood | + | + |
| 38 | bovine | blood | - | - |
| 39 | bovine | blood | + | + |
| 40 | bovine | blood | - | - |
| 41 | bovine | blood | - | - |
| 42 | bovine | blood | + | + |
| 43 | bovine | blood | + | - |
| 44 | bovine | blood | - | - |
| 45 | bovine | blood | + | + |
| 46 | bovine | blood | - | - |
| 47 | bovine | blood | + | - |
| 48 | bovine | blood | - | - |
| 49 | bovine | blood | - | - |
| 50 | bovine | blood | + | + |
| 51 | bovine | blood | - | - |

**Table 3 Cohen's kappa coefficient**

| **Summary of Case Handling** | | | | | | |
| --- | --- | --- | --- | --- | --- | --- |
|  | Individual case | | | | | |
|  | effective | | lack | | total | |
|  | N | percentage | N | percentage | N | percentage |
| PCR * ERA | 51 | 100.0% | 0 | 0.0% | 51 | 100.0% |

| **PCR * ERA crosstabs** | | | | |
| --- | --- | --- | --- | --- |
| count | | | | |
|  | | ERA | | total |
|  |  | .00 | 1.00 |  |
| PCR | .00 | 36 | 3 | 39 |
|  | 1.00 | 0 | 12 | 12 |
| total | | 36 | 15 | 51 |

| **Symmetric measurement** | | | | | |
| --- | --- | --- | --- | --- | --- |
|  | | price | Asymptotic Standard Error^a^ | approximate T^b^ | Asymptotic significance |
| Protocol measurement | Kappa | .850 | .083 | 6.137 | .000 |
| Number of effective cases | | 51 |  |  |  |

| a. No null hypothesis assumed. |
| --- |
| b. Using asymptotic standard error under the assumption of null hypothesis. |

# 2 Supplementary Figures


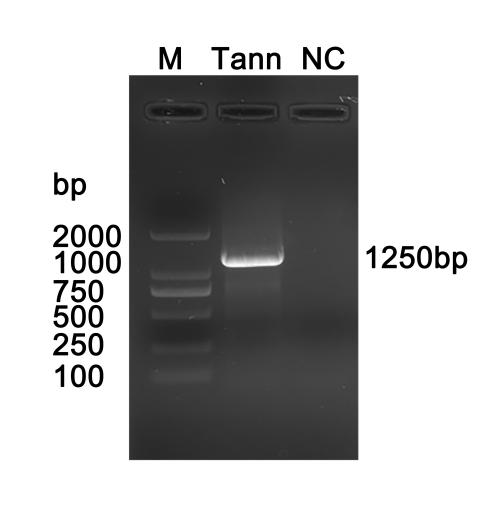


**Figure 1.** **Standard plasmid electrophoresis results of *T. annulata-enolase*.** M: Marker; Tann: enolase plasmid of bovine *Theileria annulata*; NC: negative control with ddH_2_O. Primer reaction system and reaction program for constructing positive standard plasmids: The reaction mixture is 25 μL, including 8.5 μL ddH_2_O, 1 μL of each forward and reverse primer, 13.5 μL PCR Mix, and 1 μL DNA. The thermal cycling program includes 5 minutes at 95 °C, followed by 35 cycles of 30 seconds at 95 °C, 60 seconds at 60 °C, 60 seconds at 72 °C, and finally extended for 5 minutes at 72 °C. The amplified product was stained with ethidium bromide on 1.5% agarose gel, and a 1250 bp band was visible under ultraviolet light.


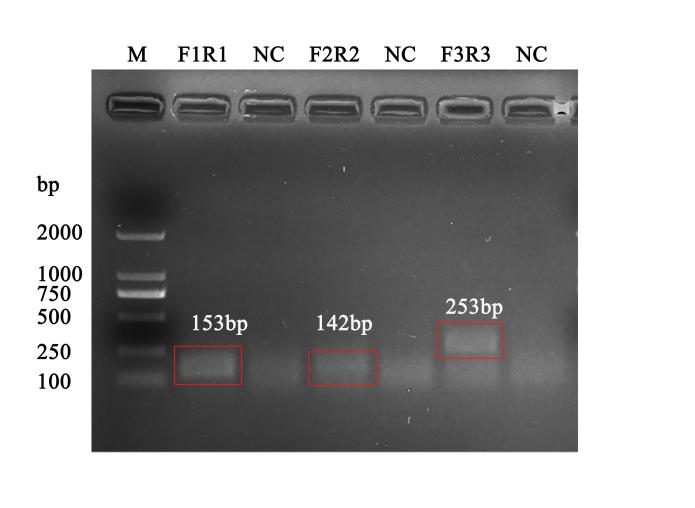


**Figure 2.** **Screening of 3 pairs of ERA primers.** ERA primer was applied to agarose gel electrophoresis after nucleic acid amplification. M: Marker; NC: negative control with ddH_2_O.


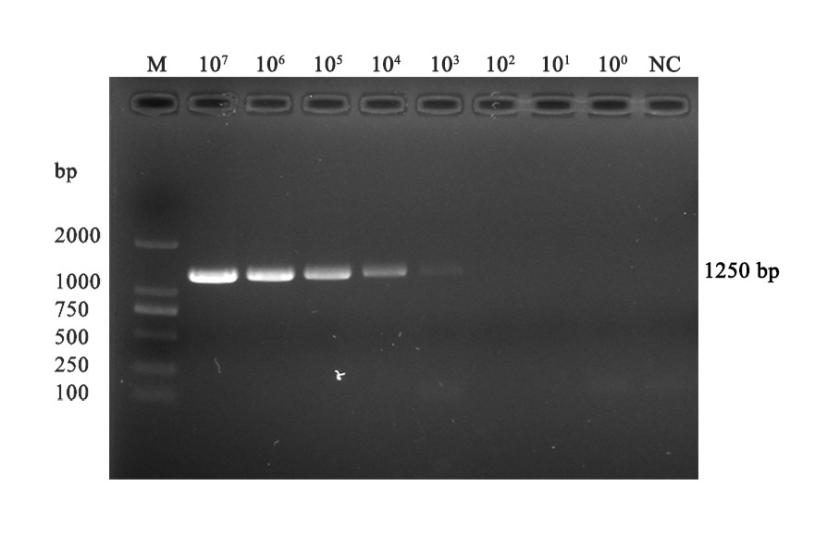


**Figure 3. Sensitivity of PCR method.** The minimum detection limit is 10^3^, and the strip is not obvious.

**
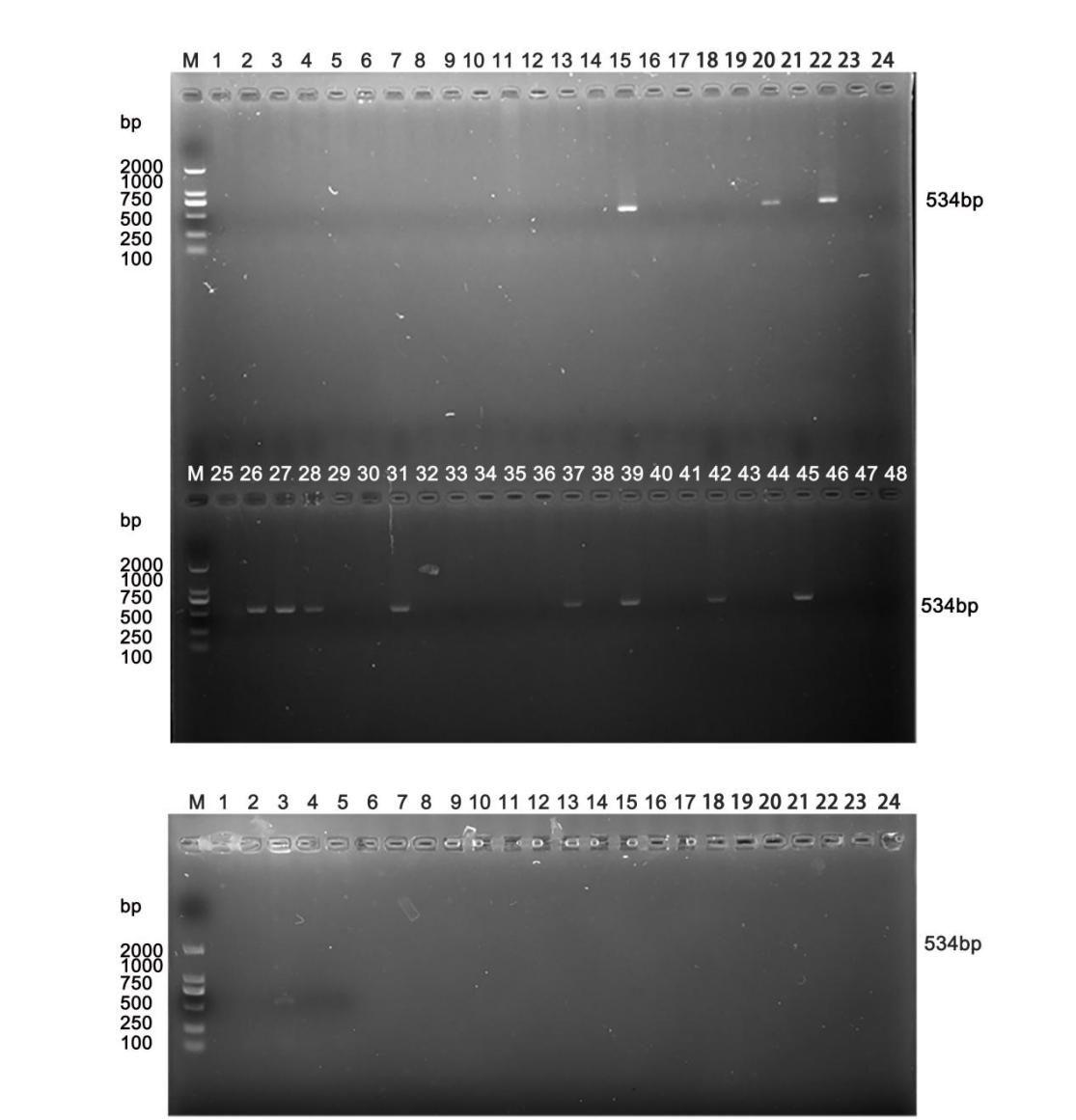
**

**Figure 4. PCR detection results of bovine blood samples.** 51 clinical samples were detected for *Theileria annulata* by PCR and analyzed by 1.5% agarose gel electrophoresis. PCR reaction conditions: 95℃ 5min, 95℃ 30s, 54.2℃ 1min, 72℃ 1min, 30 cycles, 72℃ 10min, 4 ℃ storage.

*T.annulata-B. taurus*


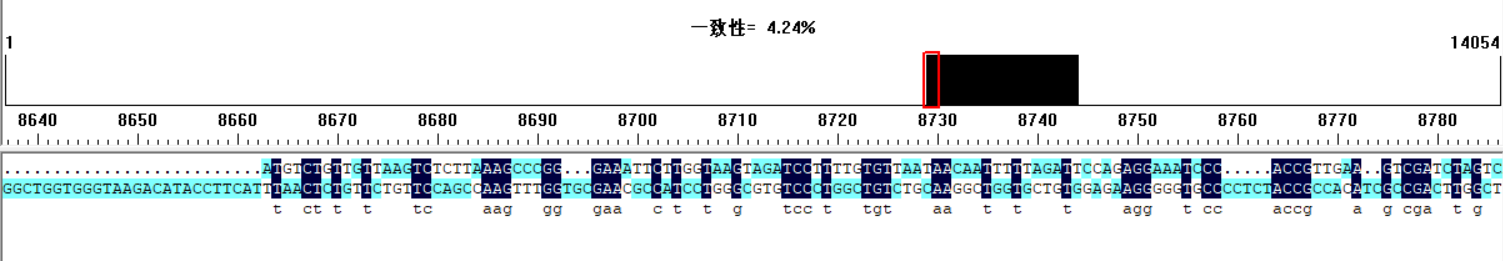


*T.annulata-B. bovis*


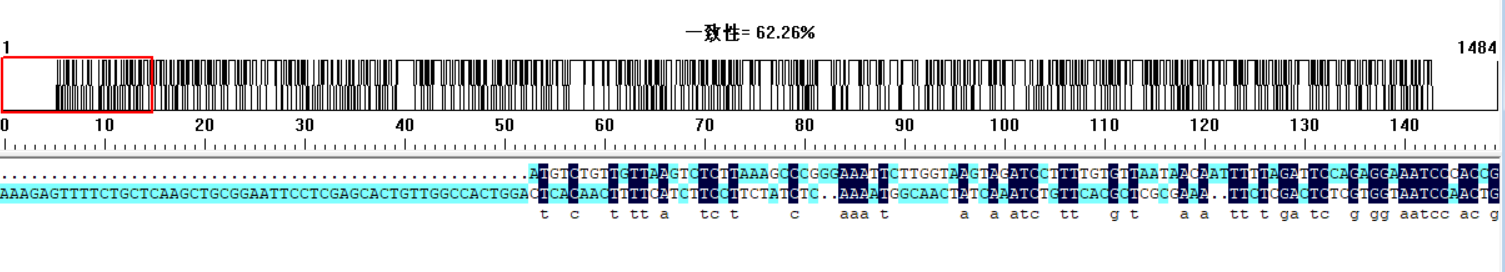


*T.annulata-A. marginale*


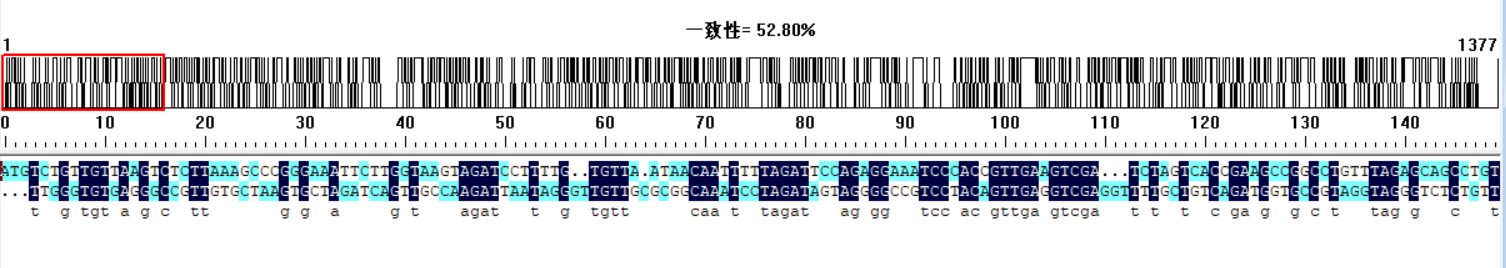


*T.annulata-Trypanosoma sp*


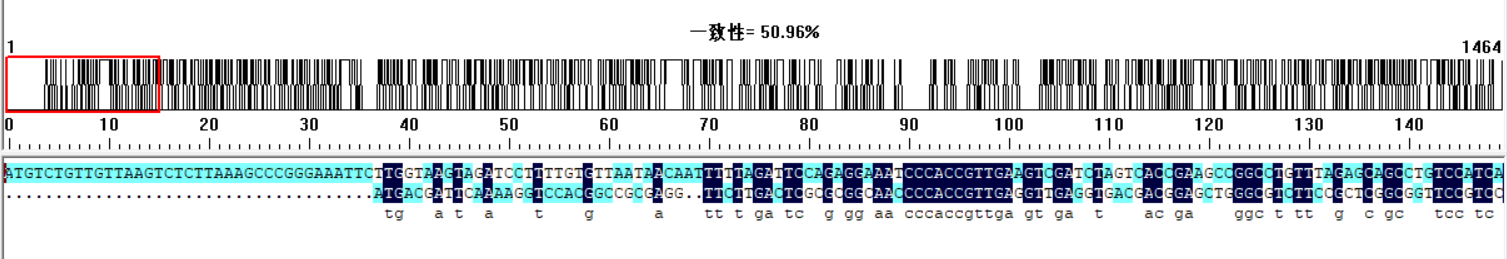


**Figure 5. Enolase gene sequence alignment results.** Using DNAMAN V6, we compared the *T. annulata* enolase sequence (HQ646253) against those of *Bos taurus* (281141), *Babesia bovis* (AK441830), *Anaplasma marginale* (7397988), and *Trypanosoma* sp. (NW_008825620).


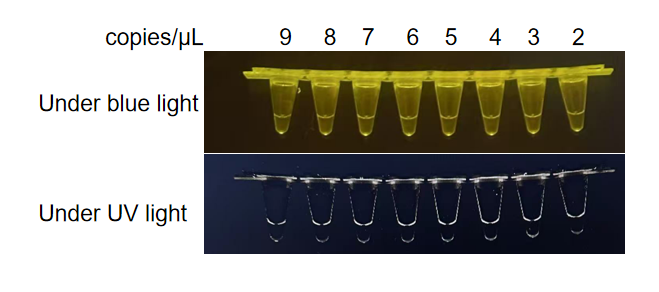


**Figure 6.** Verification testing confirms that the ERA/CRISPR-Cas12a detection method cannot detect plasmid standards with concentrations as low as 2-9 copies. Therefore, the detection limit of this method is 10 copies/μL.

**3 Supplementary Data**

**3.1 Sequencing results of plasmids:**

CCCCCGAAAGCCCGGGCCTGGTTTAGAGCAGCCTGTCCATCAGGAGCCTCAACTGGAATTTATGAAGCCTTAGAATTGCGAGATGGTGACAAAAGCCGTTATTTGGGTAAAGGTGTACTGAAAGCAGTTGAAAATGTTAACACAGTTGTGAAAAATGCAGTTGTAGGATTCGATACCTTGAATCAGAAGGAATTAGATACCTTGATGGTGCAGAAATTGGACGGCACGCAGAATGAATGGGGATACTGTAAGTCAAAACTTGGAGCAAACGCAATCCTGGTAGTATCAATGGCAGCAGCCAGAGCAGCAGCAGCCAAAAAGGGAGTCCCGCTTTACGTTCATCTAGCTCAACTGGCAGGAAAACCCACAGACAGATTTGTATTGCCAGTACCATGCTTGAACGTCATTAACGGAGGATCACACGCCGGAAACAGCCTGGCTATGCAAGAGTTTATGATTCTGCCAACGGGAGCCAACACGTTTAGAGAAGCATTACAAATGGGGGCAGAAGTTTATCACACCCTGAAATCGGTCATAAAGAAGAAGTATGGCCAAGACGCAACAAACGTAGGAGATGAAGGAGGCTTTGCACCCAATATCAAATCAGCAGAAGAGGCACTAGATCTCCTAGTTGATGCAGTTAAAAAGGCTGGATTCGAAGGGAAAGTCAATTTTGCCATGGATGTCGCAGCATCAGAATTCTATGCGAAAGAGAAGTCATCATACAACCTCGGATTCAAATGCGAAAAGGAACTCCTGAAAACTGGAGACGAAATGGTTGAATATTACACAAACCTGTGTGAAAAATACCCAATTGTTTCCATAGAGGATCCTTTCGACCAAGATGGCTGGGAATGCTACAATAAGCTTACGTCAAAGCTTGGACAAAAGGTCCAGATTGTGGGAGATGATCTTCTCGTTACAAACCCCAAGAGGATTCAAACAGCATTGGAAAAGAAGGCTTGCAACGCACTTTTGCTCAAAGTAAATCAGATTGGATCAGTGACTGAGTCAGTTGAAGCTTGCTTGCTGCCCACAAAACAATGGGAGTCATGCTTCACATAGATCAGTGAACTGAGACACATTTATGCAGATTTGTGCTGGACTTCAACAGTCAATCAAAACAGGAGCCACCTTGCAGGAGTTG


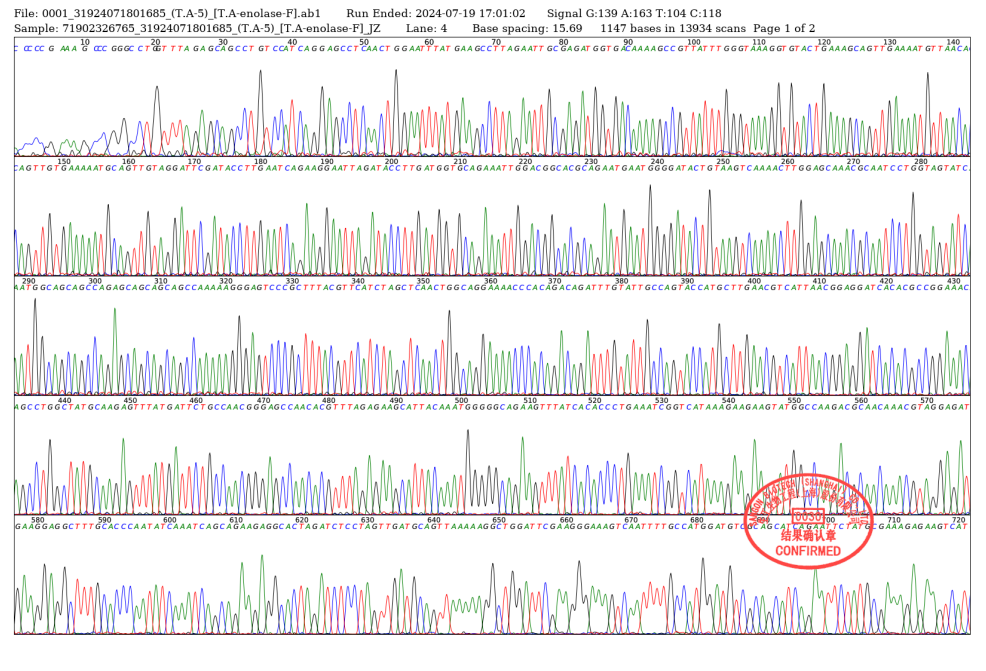


**3.2 Observe the test results using a portable blue light lamp**


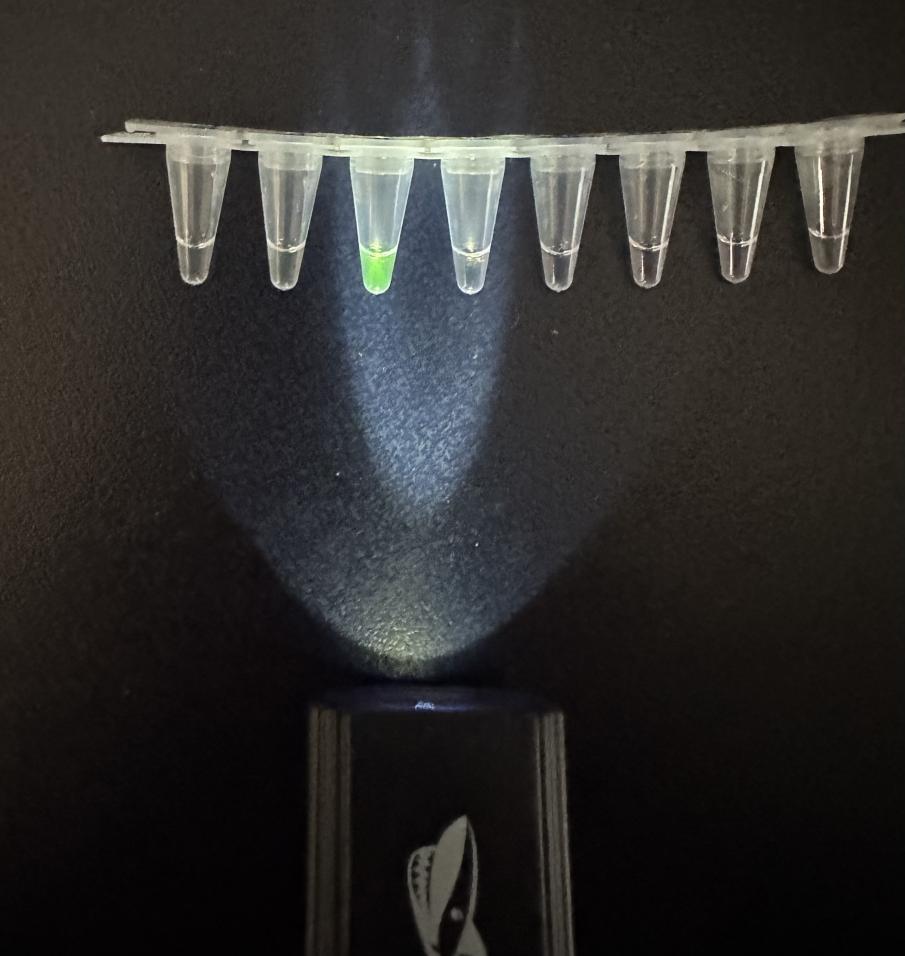


**3.3 Observe the detection results in the blue light glue cutting instrument**


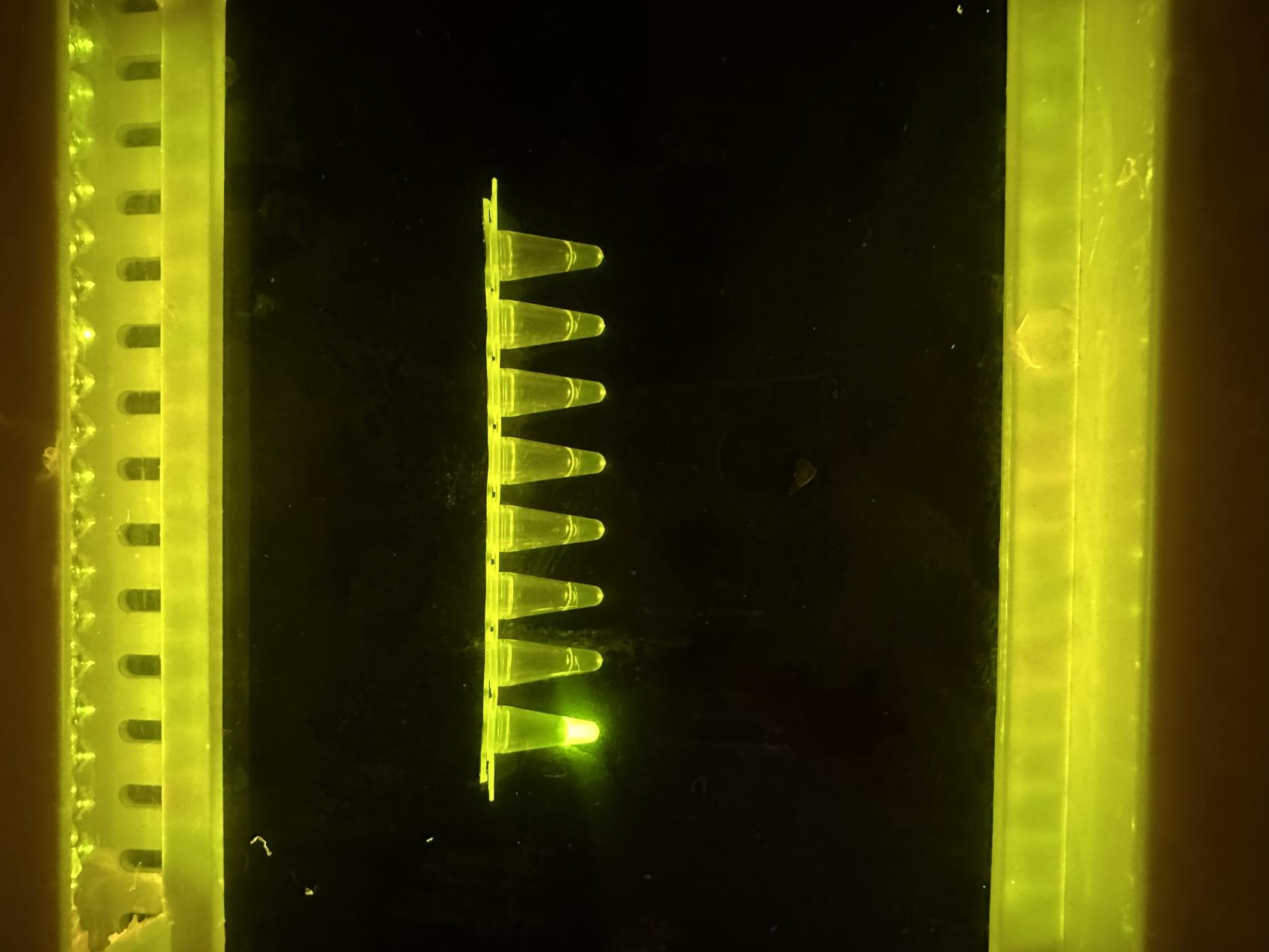


**3.4 Observe the test results under the ultraviolet gel imager**


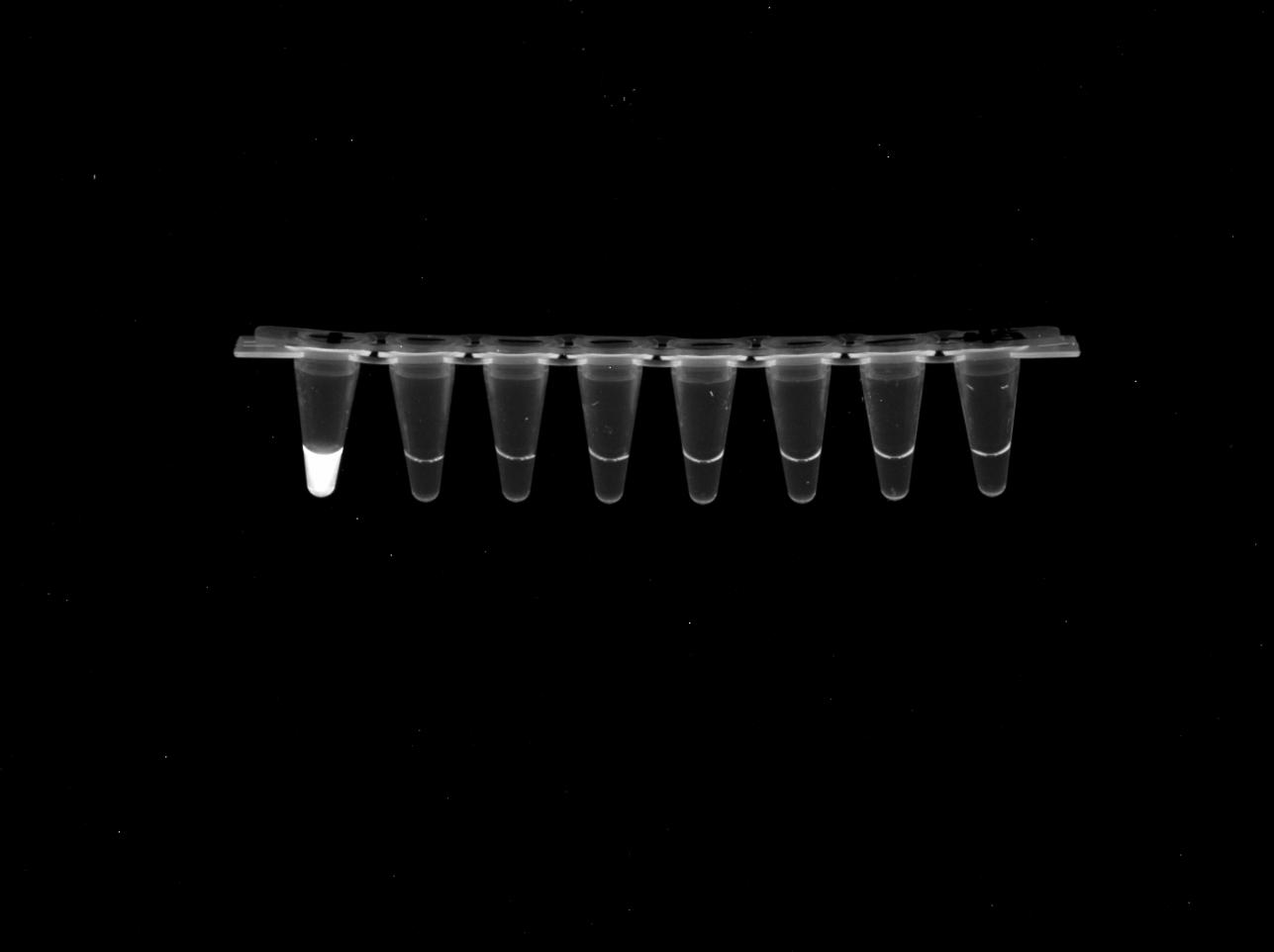

Supplement: SUPPLEMENTARY TABLE S1 — Primer and probe sequences in this study. [file Data_Sheet_1.docx]
